# Prion protein gene (*PRNP*) variation in German and Danish cervids

**DOI:** 10.1186/s13567-024-01340-8

**Published:** 2024-08-02

**Authors:** Sonja Ernst, Agata Piestrzyńska-Kajtoch, Jörn Gethmann, Małgorzata Natonek-Wiśniewska, Balal Sadeghi, Miroslaw P. Polak, Markus Keller, Dolores Gavier-Widén, Katayoun Moazami-Goudarzi, Fiona Houston, Martin H. Groschup, Christine Fast

**Affiliations:** 1 Institute of Novel and Emerging Infectious Diseases, Friedrich-Loeffler-Institut, Greifswald, Isle of Riems Germany; 2 Institute of Epidemiology, Friedrich-Loeffler-Institut, Greifswald, Isle of Riems Germany; 3 National Research Institute of Animal Production, Balice, Poland; 4 Department of Virology, National Veterinary Research Institute, Pulawy, Poland; 5 Swedish Veterinary Agency (SVA), Uppsala, Sweden; 6 INRAE, AgroParisTech, GABI, University Paris-Saclay, Jouy-en-Josas, France; 7 Division of Immunology, The Roslin Institute, Royal Dick School of Veterinary Studies, University of Edinburgh, Edinburgh, UK

**Keywords:** Chronic wasting disease, deer, *PRNP*, genotype, Germany, polymorphism, red deer, roe deer, sika deer, fallow deer, cervid, Denmark

## Abstract

The structure of cellular prion proteins encoded by the prion protein gene (*PRNP*) impacts susceptibility to transmissible spongiform encephalopathies, including chronic wasting disease (CWD) in deer. The recent emergence of CWD in Northern European reindeer (*Rangifer tarandus*), moose (*Alces alces alces*) and red deer (*Cervus elaphus*), in parallel with the outbreak in North America, gives reason to investigate *PRNP* variation in European deer, to implement risk assessments and adjust CWD management for deer populations under threat. We here report *PRNP*-sequence data from 911 samples of German red, roe (*Capreolus capreolus*), sika (*Cervus nippon*) and fallow deer (*Dama dama*) as well as additional data from 26 Danish red deer close to the German border and four zoo species not native to Germany. No *PRNP* sequence variation was observed in roe and fallow deer, as previously described for populations across Europe. In contrast, a broad *PRNP* variation was detected in red deer, with non-synonymous polymorphisms at codons 98, 226 and 247 as well as synonymous mutations at codons 21, 78, 136 and 185. Moreover, a novel 24 bp deletion within the octapeptide repeat was detected. In summary, 14 genotypes were seen in red deer with significant differences in their geographical distribution and frequencies, including geographical clustering of certain genotypes, suggesting “*PRNP*-linages” in this species. Based on data from North American CWD and the genotyping results of the European CWD cases, we would predict that large proportions of wild cervids in Europe might be susceptible to CWD once introduced to naive populations.

**Supplementary Information:**

The online version contains supplementary material available at 10.1186/s13567-024-01340-8.

## Introduction

Diseases caused by prions belong to the transmissible spongiform encephalopathies (TSEs). While cellular prion proteins (PrP^C^) naturally occur in the plasma membrane of cells in vertebrates, the infectious conformer (PrP^Sc^) stimulates PrP^C^ to misfold, resulting in accumulation of PrP^Sc^ eventually leading to invariably fatal neurodegeneration of infected hosts [1].TSEs affect both, humans (e.g. Creutzfeldt-Jakob-Disease, CJD) and animals. Bovine spongiform encephalopathy (BSE) originally found in cattle is a zoonotic pathogen, leading to variant CJD (vCJD) in humans [2]. Alongside other animal prion diseases such as scrapie in small ruminants or camel prion disease, to date, chronic wasting disease (CWD) is probably the most contagious TSE. After its first description in a mule deer (*Odocoileus hemionus*) in 1967, it took nearly a decade to identify CWD as a TSE [3]. Since then, it has spread widely across the North American continent. At the time of writing, the endemic region includes 34 US states and four Canadian provinces (May 2024) with a prevalence reaching up to 30% in free-ranging deer and in severe cases up to 90% in captive herds. Prevalence can increase up to 38% annually in captive herds [4]. Imports of infected elk (*Cervus elaphus nelsoni*) accelerated the spread of the disease to Canada and led to the introduction of CWD to South Korea [5, 6].

When the first case of CWD in a Norwegian reindeer was confirmed in March 2016 [7], the European Union introduced an active surveillance system in countries with native reindeer and moose populations (Regulation 2017/1972), namely Sweden, Poland, Finland, Iceland, Estonia, Lithuania and Latvia. Norway voluntarily participated in this regulation. This has led to the identification of 21 reindeer and three red deer from Norway as well as 18 moose from Norway, Sweden and Finland (May 2024) [8–10]. While the disease found in reindeer is unique with regard to neuropathological and biochemical characteristics, its infectivity and tissue distribution is similar to North American cases. In contrast, the affected moose and red deer cases revealed characteristics of age-dependent sporadic forms [11, 12]. Subsequent studies showed that none of these cases suggested an introduction of the North American CWD to Europe but the emergence of novel prion variants [13].

CWD prions can propagate in several deer species of subfamilies *Cervinae* and *Capreolinae*, together forming the family *Cervidae*. Species belonging to the subfamily of the *Capreolinae* affected by CWD are white-tailed deer (*Odocoileus virginianus*), black-tailed deer (*Odocoileus hemionus columbianus*), a subspecies of mule deer, reindeer (*Rangifer tarandus*) and moose (*Alces alces*) [14]. While these species are native to North America, European moose (*Alces alces alces*) and reindeer are also native to northern Europe. Small local populations of white-tailed deer, originally introduced from North America, also occur in northern Europe. Susceptible species of the subfamiliy *Cervinae* include the North American native Rocky Mountain elk (*Cervus elaphus nelsoni*), wapiti (*Cervus elaphus canadensis*) and muntjac deer (*Muntiacus reevesi*), as well as European red deer (*Cervus elaphus elaphus*) and sika deer (*Cervus nippon*) [14]. In previous studies on fallow deer (*Dama dama*), disease occurred after experimental intracerebral inoculation but in milder forms than observed in naturally infected white-tailed deer and wapiti. However, no cases were detected after natural exposure (i.e. co-grazing with infected deer) [15]. As yet, there are no data available on the susceptibility of roe deer (*Capreolus capreolus*) to CWD, the most abundant and widely distributed cervid in Europe, which plays an important role for venison production.

It is known from scrapie in sheep and goats that variations within the open reading frame (ORF) of the prion protein gene (*PRNP*), encoding for the protein structure, have a profound effect on TSE susceptibility, leading to successful breeding programs for scrapie resistance in the European Union [16, 17]. Implementing this knowledge into CWD research, several cervid *PRNP* polymorphisms within the ORF have been found which appear to alter susceptibility to CWD. For example in Rocky Mountain elk the methionine (M) to leucine (L) exchange at codon 132 (M_132_/L_132_) most probably has protective properties according to studies in Rocky Mountain elk and transgenic mice [18, 19]. Studies in white-tailed deer found 23 *PRNP* positions with single nucleotide polymorphisms, of which four non-synonymous substitutions, H_95_, (histidine), S_96_ (serine), G_116_ (glycine) and K_226_ (lysine), are associated with prolonged incubation periods and reduced clinical signs [20, 21]. However, none of these variants provide complete CWD-resistance. In European red deer, codon 226 shows a non-synonymous substitution from glutamine (Q) to glutamate (E) (Q_226_/E_226_). While experimental infection of red deer and transgenic mice with North American CWD isolates suggest no protective character of neither E_226_/E_226_, Q_226_/E_226_ or Q_226_/Q_226_ [22], previous studies in mouse models show that the amino acid at codon 226 influences the propagation of moose prions, hence disease onset and severity [23, 24]. Non-synonymous substitutions in red deer of which the influence on CWD susceptibility is yet to be determined, are G_59_/S_59_ (glycine to serine), T_98_/A_98_ (threonine to alanine), P_168_/S_168_ (proline to serine), M_208_/I_208_ (methionine to isoleucine) and I_247_/L_247_ (isoleucine to leucine), with G_59_/S_59_, M_208_/I_208_ and I_247_/L_247_ only found in single individuals [14].

Recent studies in both roe deer and fallow deer found no variations in the sequence of the *PRNP* open reading frame (ORF), with the exception of a silent mutation at codon 24 in two Swedish roe deer [25]. Whilst roe deer are homologous for wildtype (wt, T_98_P_168_Q_226_I_247_), all tested fallow deer encode the E_226_ and an additional serine to asparagine substitution at codon 138, leading to the haplotype N_138_E_226_, which is considered wildtype for fallow deer [14, 26].

In Germany, hunting is regulated by national and federal law, resulting in differently organized hunting management practices in each Federal State. In general, private lands are managed by hunting associations compiled of the landowners, while hunting on public grounds is organized by the responsible national forestry and its subdivisions. For each hunting ground and season, shooting quotas per game species are defined, which thereby are binding and need to be fulfilled. In contrast, Danish hunting law is regulated on national level only. The competent authority is the Ministry for Environmental Affairs, which hosts a “Wildlife Management Council” that includes, among others, representatives of the Danish hunting association which include about 900 local hunting clubs. Hunting grounds are in general private.

To date, there is no detailed knowledge on *PRNP* variation in middle European cervid populations. However, this information will be crucial to estimate the putative susceptibility of the indigenous deer populations for CWD and will help implementing risk assessment and surveillance strategies. With this study we therefore aimed to describe the protein-coding sequence of *PRNP* and its variation among German and Danish most important deer species, namely red, roe, sika and fallow deer, as well as non-indigenous deer species in smaller numbers. In addition, we analyzed the geographical distribution of *PRNP* variation to define possible “*PRNP-*linages”.

## Materials and methods

### German sample collection

For a better geographical overview, we divided Germany into 12 regions along the borders of the 16 Federal States by combining Rhineland-Palatinate and Saarland to region 3, Bremen and Lower Saxony to region 8, Berlin and Brandenburg to region 10 and Hamburg and Schleswig–Holstein to region 11 (Additional file 1). The majority of the samples were provided by deer stalkers of German hunting associations, its subdivisions and forestry authorities, or were collected by us in cooperation with foresters and hunters at hunting events during the hunting season 2021/22, with only a few samples from road kills or animals culled because of disease being sent in by pathologists. Stakeholders collecting samples for this study were from both private and public hunting organizations as mentioned above. Frozen samples consisted of small pieces of kidney, spleen, liver, brain, heart or muscle tissue and were archived at − 20 °C. Species determination of the sampled animals was performed by deer stalkers based on phenotype only. Additional information on sex and the estimated age were recorded, if provided. The location of the shot animals was noted at county level. We excluded 26 samples due to inconclusive results after further species determination and 47 samples which were sent in without any information on the species, leading to 3084 samples of four different deer species inhabiting Germany. All fallow deer (*n* = 8) and all but one roe deer (*n* = 2225), were free ranging. Seven of the 803 red deer and eight of the 48 sika deer were farmed animals. Four additional samples from deer of different species, not native to Germany, were sent in by zoos: one each from reindeer, white-tailed deer (WTD), Bactrian deer (*Cervus hanglu bactrianus*) and Père David’s deer (*Elaphurus davidianus*). They were genotyped and included in the study but not used for statistical analysis.

For initial analysis, we aimed to identify genotypes with at least 1% occurrence in the population (confidence interval 95%). Therefore, we had to investigate 300 samples per species. To get a countrywide overview, we took at least 25 samples per region of red and roe deer. In the case of sika deer (*n* = 39), fallow deer (*n* = 8), and zoo animals (*n* = 4), we tested all animals, as less than 25 samples were available. Due to poor sample quality, we did not obtain results for all tested animals, reducing the number of genotyped sika deer, red deer from region 1 and 5 as well as roe deer from region 4 and 7, eventually leading to 39 sika, 257 red and 311 roe deer analyzed.

In a second step it was decided to only proceed with red deer samples as no variations were seen with roe deer. Therefore, another 270 red deer samples were sequenced, which were as equally distributed across the defined regions as possible. In total, 527 red deer samples were genotypically analyzed.

Table 1 and Additional file 2 present an overview of both sample sizes and work flow.

**Table 1:** **Collected and genotyped samples per region and species in Germany and Denmark**

Region	Fallow	Red	Roe	Sika	Total
Germany					
1	1 (1)	21 (18)	225 (25)	1	248
2	–	144 (105)	174 (26)	23	341
3	–	129 (63)	272 (25)	–	401
4	–	118 (48)	122 (22)	–	240
5	–	15 (14)	113 (25)	–	128
6	–	63 (50)	104 (25)	–	167
7	1 (1)	125 (52)	159 (23)	1	286
8	–	45 (40)	343 (28)	–	388
9	–	32 (31)	97 (26)	–	129
10	5 (5)	39 (38)	319 (32)	–	363
11	1 (1)	37 (35)	128 (29)	23	189
12	–	35 (33)	169 (25)	–	204
Denmark					
13	–	31 (26)	–	–	31
Total	**8 (8)**	834 (553*)	**2225 (311)**	**48 (39)**	3115 (911)

Fallow = fallow deer (*Dama dama*); Red = red deer (*Cervus elaphus*); Roe = roe deer (*Capreolus capreolus*); Sika = Sika deer (*Cervus nippon*); 1 = Baden-Wurttemberg; 2 = Bavaria; 3 = Rhineland-Palatinate and Saarland; 4 = Hesse; 5 = Thuringia; 6 = Saxony; 7 = North Rhine-Westphalia; 8 = Bremen and Lower Saxony; 9 = Saxony-Anhalt; 10 = Berlin and Brandenburg; 11 = Hamburg and Schleswig–Holstein; 12 = Mecklenburg Western Pomerania, the number in brackets shows the number of genotyped animals; *German red deer were analysed in two steps: 257 animals in the first and further 270 animals in the second step.

### Danish sample collection

DNA samples were provided of 31 Danish red deer from the Frøslev Plantage and forest areas of Bommerlund near the German border, sampled in the years 2006 to 2009. No additional data on estimated age, sex or exact sampling location were available. Five samples had to be excluded due to poor DNA quality, leading to a total of 26 genotyped samples (Table 1, Additional file 2).

### Species determination

Species determination based on genetic marker *cytochrome B* was performed for 48 samples as results after genotyping were incompatible with anamnestic data given on species by the hunters. The same genomic DNA sample as used for *PRNP* sequencing was used to perform a PCR for amplification of the *cytochrome B* gene as previously reported [27] using GoTaq® G2 Flexi polymerase (Promega) and PCR conditions: An initial melting process at 94 °C for 3 min was followed by 40 cycles of 94 °C melting for 30 s, annealing at 47 °C for 30 s and elongation at 72 °C for 1 min with a final extension phase at 72 °C for 10 min. Successfully amplified PCR products were sent to Eurofins Genomics GmbH (Ebersberg, Germany) along with the reverse primer for sequencing. Using the Blast algorithm [28], *cytochrome B* sequences were compared with GenBank. However, for 37 samples results remained ambiguous and were sent to Laboratory of Molecular Genetics (LGM; National Research Institute of Animal Production, Poland) for further clarification. At LGM, three different methods were used: In a first step the standard PCR–RFLP for species determination and roe-deer specific PCR were performed followed by two sequencing protocols for two different fragments of the *cytochrome B* gene. The PCR–RFLP method was carried out as previously described [29] and modified with HotStarTaq DNA Polymerase (Qiagen, Venlo, Netherlands). Primers for short fragment (195 bp) of *cytochrome B* gene*,* restriction enzyme (TSP509; New England Biolabs, Ipswich, Massachusetts) and 2.5% agarose gel were used. This method allows to distinguish cattle, sheep, goat, roe deer and red deer. Since only some samples gave unambiguous results in the PCR–RFLP method, all PCR reaction products were sequenced (second method) and analysed as described below. The samples which gave electrophoresis pattern specific for roe deer, were additionally analysed by using a roe deer-specific PCR method [30]. The obtained results were confirmed by the third method: sequencing of a 472 bp fragment of *cytochrome B* gene with mcb398 and mcb869 primers as described by Verma and Singh [31]. This method was carried out with HotStarTaq DNA Polymerase (Qiagen, Venlo, Netherlands) in a 25 µL reaction volume. PCR conditions were 95 °C for 15 min followed by 35 cycles of 95 °C for 45 s, 51 °C for 1 min, 72 °C for 2 min with the final elongation at 72 °C for 10 min. All PCR products (195 bp and 472 bp) used for sequencing were electrophoresed in 2% agarose gel, purified with EPPiC (A&A Biotechnology, Gdansk, Poland), sequenced separately for each primer (same as for PCR) with BigDye™ Terminator v3.1 Cycle Sequencing Kit (Thermo Fisher Scientific, Waltham, Massachusetts, USA) and purified again with BigDye XTerminator™ Purification Kit (Thermo Fisher Scientific, Waltham, Massachusetts, USA). All kits were used according to manufacturer’s protocols. The sequences were read in Genetic Analyzer 3500×l and edited in BioEdit Sequence Alignment Editor [32]. Sequences for both fragments were analysed with BLAST and compared to reference mtDNA genomes, *cytochrome B* gene sequences (GeneBank) and to BLAST nucleotide database. Reference sequences are given in Additional file 3.

### *PRNP* sequencing of German and Danish samples

Genomic DNA of German samples was isolated using the QIA^®^ DNA Mini Kit (Qiagen, Oslo, Norway) according to manufacturer’s instructions. The DNA of all Danish samples was already prepared for PCR. Amplification of the open reading frame (ORF) of cervid *PRNP* was performed following the PCR protocol and primers Ce_-143d (ATG​GAA​TGT​GAA​GAA​CAT​TTA​TGACCTA) and Ce_ + 139u (TAA​GCG​CCA​AGG​GTA​TTA​GCAT) as recently described [26], using Phusion High Fidelity DNA polymerase (Thermo Fisher Scientific, Waltham, Massachusetts, USA). Adjusting the initialization time as well as annealing time and temperature improved the target amplification for German and Danish samples, leading to PCR conditions: Initial 10 min at 95 °C, followed by 40 cycles of 95 °C for 35 s, 58 °C for 35 s and 72 °C for 1 min with a final 10 min phase at 72 °C. After purification of the amplified PCR fragments using the QIAquick^®^ Nucleotide Removal Kit (Qiagen, Oslo, Norway) according to manufacturer’s instructions the samples were send to Eurofins Genomics GmbH (Ebersberg, Germany) along with forward and reverse primer pairs Ce_ + 70u (GCT​GCA​GGT​AGA​TAC​TCC​CTC) and Ce19_F (ATT​TTG​CAG​ATA​AGT​CAT​C) or Ce19_F and Ce778_R (AGA​AGA​TAA​TGA​AAA​CAG​GAAG) or Ce778_R and Ce08_F (ACACCCTCTTTATTTTGCAG) for the samples with the 24 bp deletion, for sanger sequencing [18]. The amplificants are about 800 bp long, containing the 771 bp long open reading frame.

Sequence data was aligned and edited using Geneious Prime 2021.0.1 and analyzed on a nucleotide and protein-based level in comparison to reference sequences with accession numbers DQ154293 (reindeer) [33], MG856905 (white-tailed deer) [34], MK103016 (roe deer) [26], MK103017 (fallow deer) [26], MK103018 (sika deer) [26], MK103027 (red deer and Bactrian deer) [26], and MW804583 (Père David’s deer) [35], as shown in Additional file 4. For white-tailed deer, reindeer and Père David’s deer other reference sequences than for red, roe and sika deer were necessary, as those species present another wildtype.

### Cloning of *PRNP* for haplotype determination

To determine the haplotype and the exact location of a 24 bp deletion, seen in 12 red deer, we cloned the relevant samples. DNA was amplified using the GoTaq^®^ G2 Flexi polymerase (Promega) and primer pair CE08_F and Ce778_R and following PCR conditions: After initial 5 min at 94 °C, 40 cycles of 30 s at 94 °C, 30 s at 58 °C and 1 min at 72 °C followed with a final 7 min at 72 °C for ligation. PCR products were purified using the PureLink™ PCR Purification Kit (Invitrogen) as described by the manufacturer. For ligation and transformation, the Topo™ TA Cloning™ Kit (Invitrogen) was used according manufacturer’s instructions. Eight colonies per sample were picked and directly amplified under following PCR conditions with primer pair M13F (GTAAAACGACGGCCAG) and M13R (CAGGAAACAGCTATGAC) and GoTaq^®^ G2 Flexi polymerase (Promega): After initial 10 min at 94 °C, 35 cycles of 94 °C for 1 min, 55 °C for 1 min and 72 °C for 1 min 10 s were followed by 72 °C for 10 min. Finally, samples were sent to Eurofins Genomics GmbH (Ebersberg, Germany) along with primer pair CE08_F and Ce778_R for sequencing both strands. Sequence data were analyzed as described before.

### Accession numbers

Eleven sequences with following accession numbers were deposited in GenBank^®^: PP512522 (roe deer), PP512523 (Bactrian deer), PP512524 (Père David’s deer), PP512525 (red deer with A_98_), PP512528 (red deer with A_98_L_247_ haplotype), PP512538 (red deer with Δ_69-77_A_98_ haplotype), PP512530 (red deer with Δ_69-77_ haplotype), PP512531 (red deer with E_226_), PP512534 (red deer wildtype), PP512536 (fallow deer) and PP512537 (sika deer).

### Statistical analysis and map development

For the statistical analysis and the map development we used the R statistical software version 4.3.0 and RStudio version 2023.03.0 and the packages readxl, sf, tmap, dplyr, purrr, tydyr, ggplot2, and EpiR (references see Additional file 5). We carried out a descriptive statistic and calculated the point estimates including confidence intervals [36] using EpiR to compare the proportion of different genotypes by species, year and region.

### Data sources

The hunting data for Germany were provided by Thünen Institut (Additional file 6) [37], the hunting data for Denmark were obtained from the website of the Aarhus University [38], the map data were obtained from the Eurostat website [39] license: © EuroGeographics for the administrative boundaries, and for the German district boundaries from the Federal Agency for Cartography and Geodesy (BKG) [40], license: Datenlizenz Deutschland Namensnennung 2.0 [41].

## Results

For genotype and haplotype nomenclature, we followed the EFSA suggestions [10] on naming alleles by codon position and a one letter amino acid abbreviation for all positions that deviate from wildtype (wt). Wt for roe, red and sika deer is T_98_P_168_Q_226_I_247._ Table 2 gives an overview of all genotypes seen in German and Danish deer.

**Table 2:**
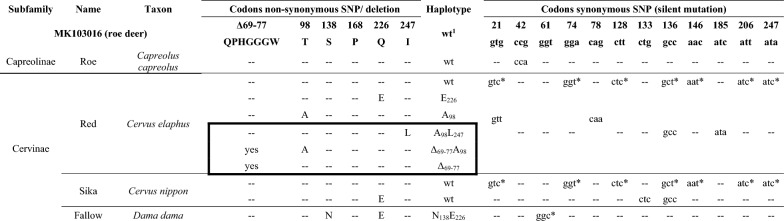
**Overview of SNPs in surveyed native German and Danish deer species**

Overview of all polymorphisms in reference to the roe deer sequence MK103016 [26]. For non-synonymous SNP/ deletion amino acids are given (left side); Haplotype is named after amino acid and *PRNP*-positions when different to wildtype; ^1^wt = wildtype/ consensus for European red, roe and sika deer with key-positions T_98_P_168_Q_226_I_247_; One-letter amino acid abbreviation is used (left side): A = alanine, E = glutamate, I = isoleucine, L = leucine, N = asparagine, P = proline, Q = glutamine, S = serine, T = threonine, Δ = Deletion; Nucleotide sequence for codon position with synonymous SNPs is given (right side): a = adenine, c = cytosine, g = guanine, t = thymine; * synonymous polymorphism in comparison to reference, does not represent an actual SNP but a species-specific alteration compared to roe deer, ^1^ wt = wildtype T_98_P_168_Q_226_I_247_; No alteration in the amino acids compared to reference are denoted with “–”; back frame denotes haplotypes solely seen in German red deer; Roe = roe deer; Red = red deer; Sika = sika deer; Fallow = fallow deer.

### Species determination

For 48 samples, species verification was performed due to inconsistencies between the phenotypic species identification provided by hunters and the *PRNP* sequence data. In 22 cases, the species identification was corrected to either red deer (*n* = 5), roe deer (*n* = 15) or European mouflon (*Ovis aries musimon*) (*n* = 2). The two mouflons were excluded from the study. The results for 26 samples were repeatedly inconclusive, so that it was not possible to determine the exact species. This includes 20 samples phenotypically identified as red deer and six phenotypically determined as roe deer. For additional 47 samples, no species information was provided at all. Those, as well as the 26 samples with inconclusive results after species identification, were excluded from further analysis of genotype frequencies.

### Red deer samples

With around 75 thousand red deer shot per season, it is one of the most important game species in Germany [42], highly valued for its venison and leather. It is also farmed for venison production and considered royalty among German wild animals. However, red deer are also highly contentious in the context of human-wildlife conflict, as they can cause costly economic damage in agriculture and forests used for timber production. This has led to the establishment of so called “red deer districts” in Germany, which are defined by federal law. Any red deer found outside these districts are culled, resulting in large areas in southern and north-western Germany that are free of this species [43] as indicated in Additional file 6.

Overall, the investigation of *PRNP* ORF sequences from 527 German and 26 Danish red deer samples revealed seven single nucleotide polymorphisms (SNPs) including four synonymous (silent) mutations (SM) at codons 21 (c/t, nucleotide position 63), 78 (g/a, nucleotide position 234), 136 (t/c, nucleotide position 408) and 185 (c/a, nucleotide position 555). The 21 c/t (*n* = 72/527) and 136 t/c (*n* = 415/527) nucleotide exchange were the most common SM in German deer. For codon 21 wildtype c/c was still the most frequent allele (*n* = 455/527) in German red deer, with only 49/72 individuals heterozygous for c/t and 23/72 homozygous for t/t. In contrast, at codon 136 the majority of German samples were heterozygous for t/c (*n* = 212/527) or homozygous for c/c (*n* = 203/527), with only 112/527 animals homozygous for the wildtype t/t. The SNP at codon 78 occurred in only a small number of German red deer (*n* = 7/527) and the SNP at codon 185 was seen in only one German individual, which was heterozygous for c/a. In Danish deer, the SM at codon 21 was more balanced, with most animals showing heterozygosity c/t (*n* = 10/26), followed by wildtype c/c (*n* = 9/26) and seven animals homozygous for t/t. At codon 136, the majority of the tested population was homozygous for c/c (*n* = 17/26), and the remaining animals (*n* = 9/26) were heterozygous for t/c.

Three non-synonymous substitutions at codons 98 (a/g, nucleotide position 292), 226 (c/g, nucleotide position 676) and 247 (a/c, nucleotide position 739) were seen in German and Danish red deer (Table 2). These led to amino acid substitutions threonine to alanine (T_98_/A_98_, 163/553), glutamine to glutamic acid (Q_226_/E_226_, *n* = 411/553) and isoleucine to leucine (I_247_/L_247_, *n* = 8/553), respectively. While the substitutions at codons 98 and 226 were seen in German and Danish red deer, the polymorphism (PM) at 247 was seen only in German red deer. Genotype analysis inferred linkage between codons 21, 136 and 226, such that nucleotide positions 63t and 408c predominantly occurred on a E_226_ background with nucleotide position 676 g. 23 of the in total 30 red deer (23 German and 7 Danish) homozygous for 63t/t were also 408c/c and 676 g/g (E_226_/E_226_) homozygous. A_98_ was found exclusively in combination with Q_226_ whereas the L_247_ substitution was solely seen in animals homozygous or heterozygous for A_98_.

In addition, a novel 24 bp deletion within the octapeptide repeat region from codon 69 to 77 (nucleotide positions 207 to 230; ccaacctcatggaggtggctgggg) was detected in 12 (*n* = 12/527) German individuals, resulting in the loss of one octapeptide (QPHGGGW). The deletion was linked to T_98_ and A_98_ resulting in haplotypes Δ_69-77_ and Δ_69-77_A_98_, with haplotype Δ_69-77_A_98_ being more frequent (*n* = 8/12) than Δ_69-77_ (*n* = 4/12).

In summary, a total of six haplotypes were seen in German and Danish red deer namely wt (T_98_P_168_Q_226_I_247_), E_226_, A_98_, A_98_L_247_, Δ_69-77_, Δ_69-77_A_98_, leading to 14 genotypes, found in the investigated red deer populations.

### Genotype frequencies and geographical distribution of red deer samples

**Figure 1:**
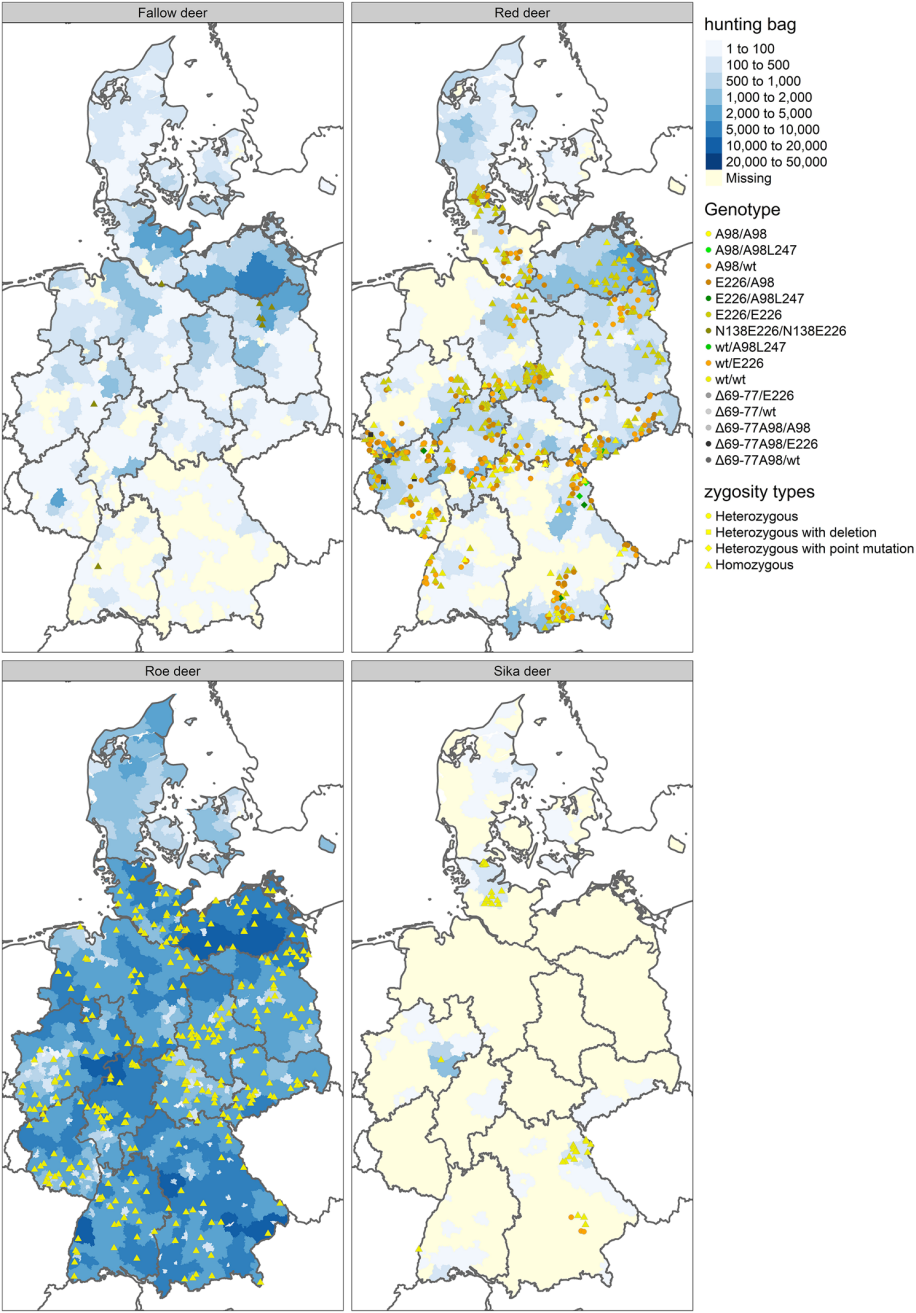
**Geographic distribution of genotypes per species.** The blue background of the map indicates the hunting bag per species on county level during the hunting season 2021/22, the darker the blue, the more animals were shot. Genotypes per species are given with different symbols and colours. Each symbol represents one sample. As no exact location but the originating county of the samples was provided, the symbols were randomly placed within the corresponding county borders. The hunting bag of fallow and red deer is comparable. Only few fallow deer were tested in this study, all were of the same genotype N_138_E_226_, which is considered wildtype for this species. Even though, red deer are not present in wide parts of north-western and southern Germany, samples of most counties with a hunting bag for red deer were tested. A broad variance of genotypes for red deer was found. Roe deer abundantly inhabit most parts of Germany apart from densely populated areas, such as big cities. The sample set tested in this study is geographically evenly distributed across Germany and homozygous for wildtype (wt). Sika deer only occurs in four larger secluded regions and a few smaller areas. Animals of the larger areas were tested as well as a small number of farmed sika deer from southern Germany (region 1), genotypic variation was only seen in these animals.

**Figure 2:**
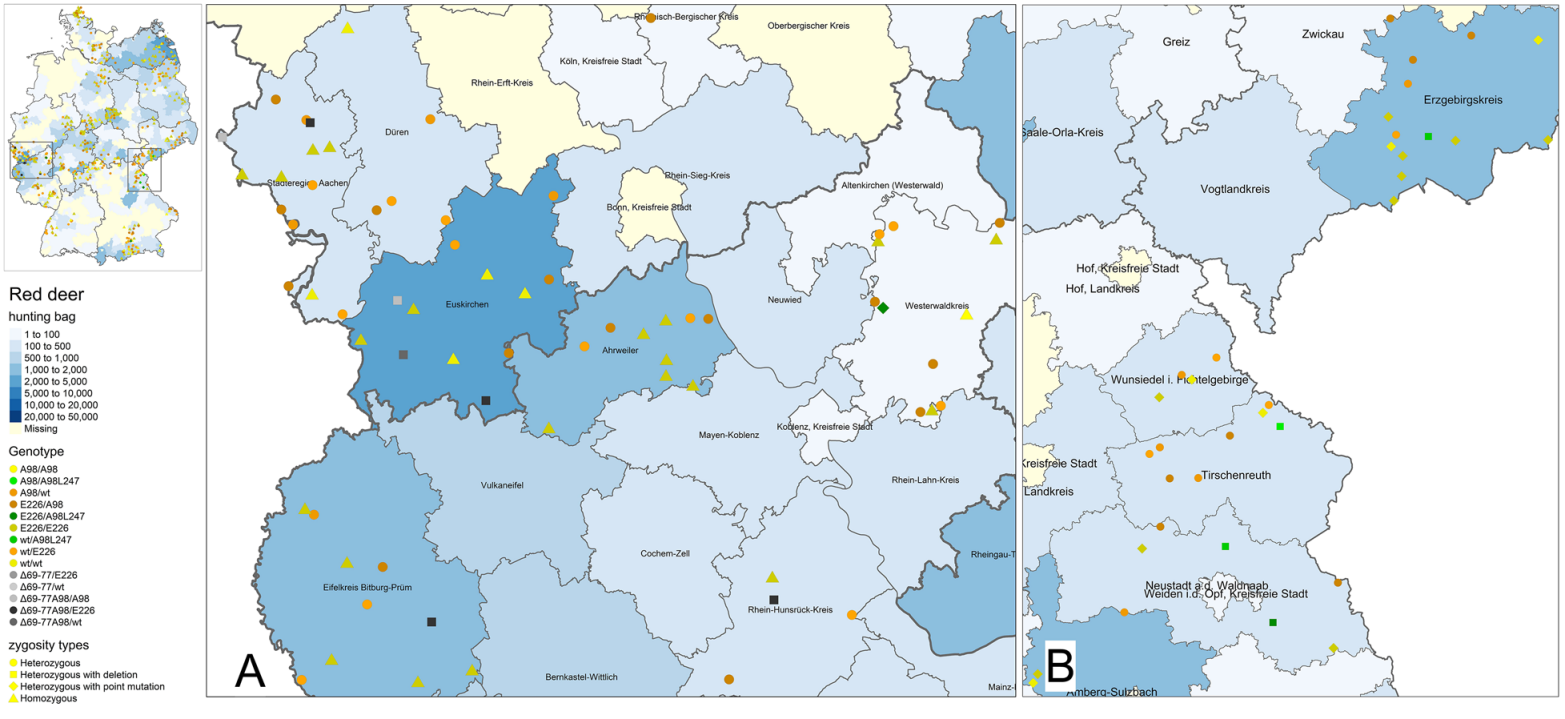
**Geographic distribution of the 24 bp deletion and I247/L247-heterozygosity clusters in red deer.** The blue background of the map indicates the number of hunted red deer on county level during the hunting season 2021/22, the darker the blue, the higher the hunting numbers. County names are given within the corresponding borders. Genotypes are marked by different colours, different zygosity types (Heterozygous, homozygous, heterozygous with point mutation) are given in different shapes. Localisation of the sampled animals was given on county level only, therefore the symbols indicating a sample were randomly placed within the borders of the corresponding county. **A** Close-up on the German-Czech border region. The different shades of green display the findings of the red deer heterozygous for I_247_/L_247_. In counties bordering the Czech Republic (Erzgebirgskreis, Tirschenreuth, Neustadt a. d. Waldnaab) most red deer with this PM were seen. **B** Close-up of the western part of Germany, were a cluster of red deer carrying the 24 bp deletion was seen. Light-grey, dark-grey and black squares display the samples found with the novel deletion. Six animals derived the German-Belgian border region, which is also in close proximity to France and Luxembourg.

The majority of genotypes in red deer (A_98_/E_226_, E_226_/E_226_, wt/A_98_, wt/E_226_ and wt/wt) were evenly distributed across Germany (Figure 1). Animals with the I_247_/L_247_ heterozygosity were almost exclusively seen in Bavaria (Region 2) and Saxony (Region 6), at the border to the Czech Republic (Figure 2A), whereas animals carrying haplotypes with the 24 bp deletion were predominantly located in the western part of Germany (Figure 2B), near the border to France, Belgium, and Luxembourg.

**Figure 3:**
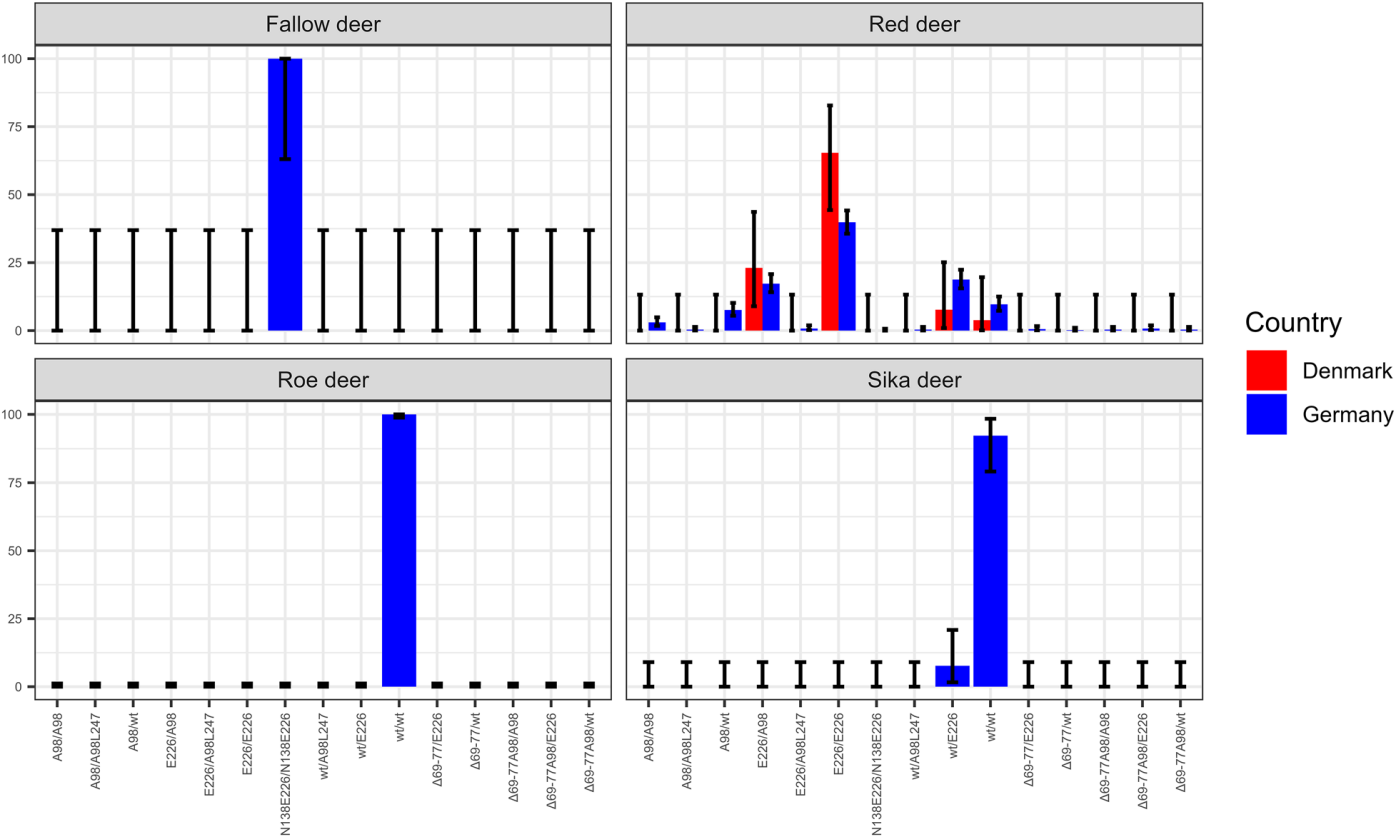
**Genotype frequencies in German and Danish wild cervids.** Confidence intervals are given in black bars. While fallow and roe deer were found homozygous, red and sika deer show *PRNP*-variability. In both species, genotype frequencies are unevenly distributed. It stands out that in red deer from Germany as well as Denmark, not wildtype (wt) is most common but E_226_ homozygosity.

**Figure 4:**
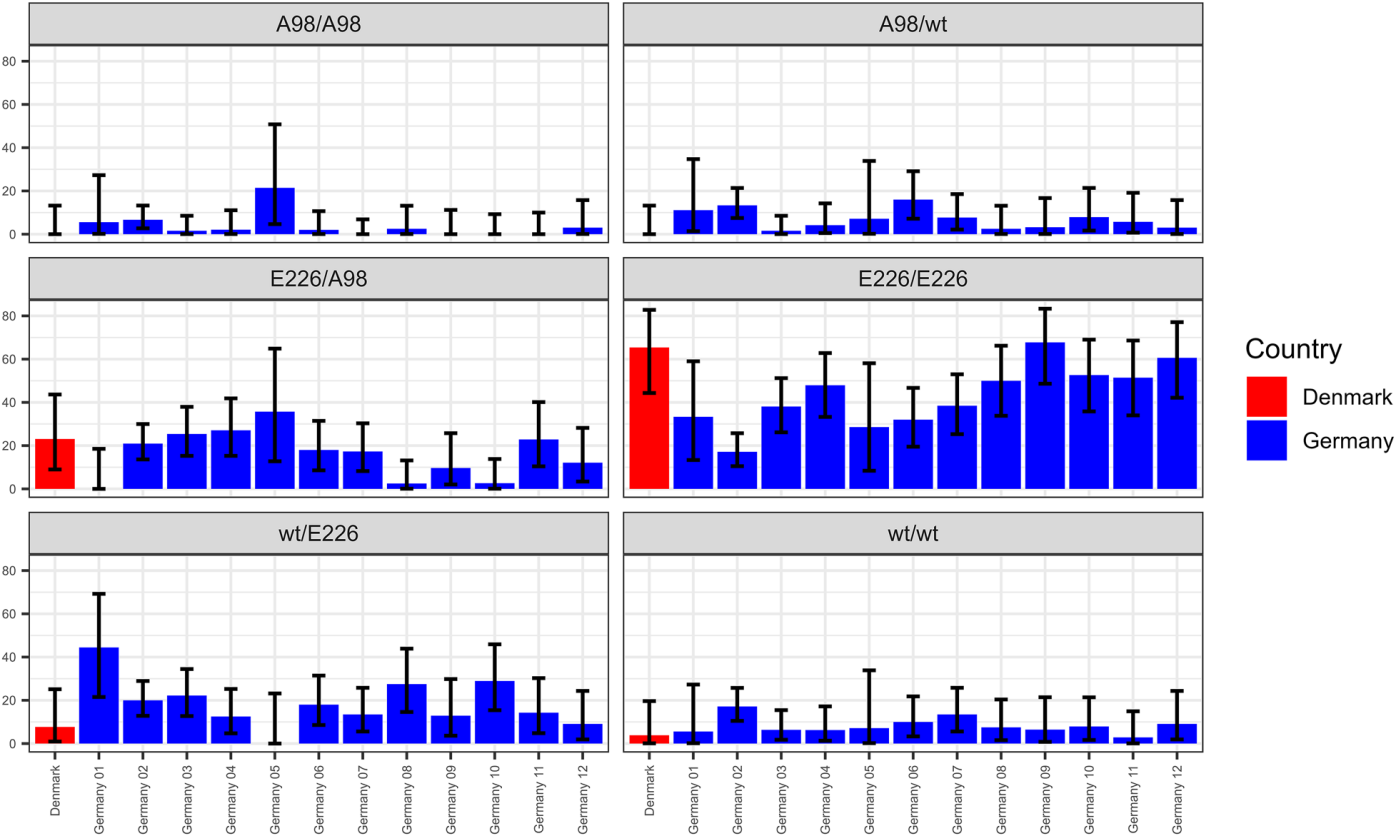
**Comparison of genotype frequencies found in German and Danish red deer.** Genotype frequencies in red deer per German region (blue) as well as the Danish subpopulation (red) are displayed. E_226_/E_226_, wt/E_226_ and A_98_/E_226_ are widely distributed throughout the population. While wildtype (wt) in region 2 (Germany 02) is significantly lesser seen than in other regions, A_98_/A_98_ tends to occur more often in the southern half, clustering in region 1, 2 and 5. However, this is statistically not significant. Black bars indicate confidence intervals. Germany 01 = Baden-Wuerttemberg; Germany 02 = Bavaria; Germany 03 = Rhineland-Palatinate and Saarland; Germany 04 = Hesse; Germany 05 = Thuringia; Germany 06 = Saxony; Germany 07 = North Rhine-Westphalia; Germany 08 = Bremen and Lower Saxony; Germany 09 = Saxony-Anhalt; Germany 10 = Brandenburg and Berlin; Germany 11 = Hamburg and Schleswig–Holstein; Germany 12 = Mecklenburg Western Pomerania.

E_226_/E_226_ (*n* = 210/527) was the most frequently detected genotype throughout Germany and also predominantly seen in the investigated Danish population (*n* = 17/26). Further homozygous animals were less frequent with only 51/527 animals of wt/wt genotype from Germany and a single Danish individual (*n* = 1/26). Just 16 deer homozygous for A_98_/A_98_ were registered (*n* = 16/527), which were mainly seen in middle Germany and the south-eastern border region, but not found in Denmark. German animals were heterozygous with high numbers of wt/E_226_ (*n* = 99/527) and A_98_/ E_226_ (*n* = 91/527), whereas only 40/527 individuals carried wt/A_98_ genotype. 6/26 Danish deer carried A_98_/E_226_ genotype, while two (*n* = 2/26) were wt/E_226_ heterozygous. The frequency of red deer with *PRNP* encoding the 24 bp deletion was very low (n = 12/527), highly variable in combination with polymorphisms and limited to German red deer. They occurred as heterozygous genotypes wt/Δ_69-77_ (*n* = 1/12), Δ_69-77_/E_226_ (*n* = 3/12), Δ_69-77_A_98_/A_98_ (*n* = 2/12), wt/Δ_69-77_A_98_ (*n* = 2/12) or Δ_69-77_A_98_/E_226_ (*n* = 4/12). No animals homozygous for the deletion were found. The I_247_/L_247_ heterozygosity (*n* = 8/527) was even rarer and also exclusively seen in German red deer, found in animals heterozygous for A_98_/A_98_L_247_ (*n* = 2/8), wt/A_98_L_247_ (*n* = 2/8) and E_226_/A_98_L_247_ (*n* = 4/8). Table 3, Figure 3 and Additional file 7 display the genotype frequencies and genotype prevalence in red deer. Figure 4 compares their distribution per region.

**Table 3:** ***PRNP*-genotype numbers in German wild Cervids and Danish cervids**

Genotype	Fallow	Red	Red DK	Sika	Roe	Total
A_98_/A_98_	–	16	–	–	–	16
wt/wt^1^	–	51	1	36	311	399
E_226_/E_226_	–	210	17	–	–	227
N_138_E_226_/N_138_E_226_	8	–	–	–	–	8
wt/A_98_	–	40	–	–	–	40
wt/E_226_	–	99	2	3	–	104
A_98_/E_226_	–	91	6	–	–	97
Δ_69-77_A_98_/A_98_	–	2	–	–	–	2
Δ_69-77_A_98_/E_226_	–	4	–	–	–	4
wt/Δ_69-77_A_98_	–	2	–	–	–	2
Δ_69-77_/E_226_	–	3	–	–	–	3
wt/Δ_69-77_	–	1	–	–	–	1
A_98_/A_98_L_247_	–	2	–	–	–	2
E_226_/A_98_L_247_	–	4	–	–	–	4
wt/A_98_L_247_	–	2	–	–	–	2
Total	8	527	26	39	311	911

Genotype is named after amino acid and *PRNP*-positions when different to wildtype; ^1^ wt = wildtype for red, roe and sika deer with key-positions T_98_P_168_Q_226_I_247_ wt = wildtype T_98_P_168_Q_226_I_247_; Red = red deer; Red DK = Danish red deer; Sika = sika deer; Roe = roe deer; Fallow = fallow deer.

### Sika deer samples

Sika deer are the least common free ranging cervid species in Germany, occurring in separated areas in south-western Baden-Wuerttemberg (region 1), eastern Bavaria (region 2), North Rhine-Westphalia (region 7), Lower Saxony (region 8) and Schleswig Holstein (region 11), resulting in a low number of about 2400 hunted animals per season [44] as shown in Additional file 6.

All 48 sika deer samples were used for genotyping. For nine samples genotyping was not successful due to poor tissue quality of the samples. One SM at codon 133 (c/g, nucleotide position 399) was detectable in four of the remaining 39 samples, exclusively in animals of wt/wt. All sikas with the SM at codon 133 were free ranging animals of the Bavarian habitat (region 2) and most were heterozygous for c/g (*n* = 3/4), with only one animal (*n* = 1/4) homozygous for c/c. The majority of Sika deer are homozygous for wt/wt (*n* = 36/39). Only three individuals (*n* = 3/39), all of the same sika deer farm in Lower Bavaria (region 2), showed heterozygosity at codon 226, resulting in the genotype wt/E_226_ (Figures 1 and 3, Tables 2 and 3).

### Roe deer

Roe deer occur in all parts of Germany in large numbers and are therefore the most frequently shot game species. Hunting statistics indicate a stable population with an increasing number of hunted animals in the last 20 years, with reporting of over 1.2 million animals shot during the 2021/22 hunting season [45]. As they can easily adapt to new environments, they also inhabit densely populated areas, yet at lower numbers (Additional file 6).

311 roe deer from all regions of Germany were genotyped for this study (Table 3). In a small number of roe deer (*n* = 5/311) of geographically distant counties in regions 3, 7, 8 and 9, a SM at codon 42 (g/a, nucleotide position 126) was seen (Table 2). All animals with this SM were g/a heterozygous at nucleotide position 126. However, no *PRNP* variation resulting in amino acid substitutions were seen in roe deer (Figure 3, Additional file 7).

### Fallow deer and zoo animals

Fallow deer are less commonly seen in the wild in Germany, with about 68 000 animals shot in 2021/22. However, they are frequently kept in captivity [46]. In total eight fallow deer were genotyped. The number of fallow deer shot in Northern Germany is higher than in the South, in several regions no fallow deer was shot in 2021/22 (Additional file 6). Most animals originated from region 10 (*n* = 5/8), with single individuals from region 1 (*n* = 1/8), region 7 (*n* = 1/8) and region 11 (*n* = 1/8) (Table 1). All were homozygous for N_138_E_226/_ N_138_E_226_ (Table 3).

Genotyping was additionally performed for four zoo animals all sent in by pathologists. While samples from one white-tailed deer (WTD), reindeer and Bactrian deer each derived from the same zoo in region 6, a sample from one Père David’s deer was sent in from a zoo in region 1. For Père David’s deer genotype S_138_I_208_E_226_ could be confirmed. The single investigated WTD in our study was identical to reference sequence with genotype Q_95_G_96_S_100_N_103_A_123_Q_226_. This study’s reindeer showed M_2_S_129_S_138_M_169_N_176_S_225_. As there was no reference sequence specifically available for Bactrian deer, the sequence was compared to a red deer sequence with L_247_. Both sequences were identical but position 247, where the Bactrian deer showed I_247_, resulting in the genotype wt/wt (T_98_P_168_Q_226_I_247_/T_98_P_168_Q_226_I_247_).

## Discussion

To our knowledge this is the first study analyzing *PRNP*-variation in wild deer species inhabiting Germany and Denmark. It is also the first to report the *PRNP*-sequence of a Bactrian deer. Including 911 samples from wild deer, this study gives a general overview over genotypes present in German red, roe, sika and fallow deer as well as a small population of Danish red deer. In total, seven haplotypes (wt, Δ_69-77_, Δ_69-77_A_98_, A_98_, A_98_L_247_, N_138_E_226_, E_226_) were found, of which six were seen in red deer, which is therefore showing the highest *PRNP* variability. Besides a novel 24 bp deletion, all polymorphisms identified in this species were previously described in Europe. In addition, we characterized the genotype of four zoo animals, including a Bactrian deer, a reindeer, a Père David’s deer and a white-tailed deer (WTD).

Several limitations associated with investigating wild animals made statistical analyses challenging. Firstly, there are no accurate estimations of population sizes. However, we were able to use the number of registered shot and roadkill animals collected in all German Federal States as well as Denmark per year, allowing us to determine approximate values. Secondly, sampling effort was uneven across Germany, leading to counties which were overrepresented due to high numbers of submitted samples while other counties could not be sampled. Furthermore, the Danish samples tested here derive from a single forest area near the German border (Frøslev Plantage, Bommerlund) and were already collected 15 years ago. Even though, the Danish samples give no insight in the current *PRNP*-genotype situation in Danish deer, there is a clear congruence to the current situation in Germany. Collecting samples was easiest and most effective during driven hunts and large hunting events, but this most probably led to accidental sampling of family groups and closely related animals. Nevertheless, randomisation of samples was achieved retrospectively by defining 13 regions of interest from which 25 equally distributed samples were chosen. Based on the variations seen in red deer samples a more targeted sample collection was subsequently performed for further investigation.

Not all anamnestic information on the species from which samples were derived was correct, resulting in the exclusion of some samples, for which the species could not be clarified. The most likely explanations for these inconclusive results are contamination during the sampling process and/or poor sample quality, leading to mixed DNA in one sample tube. This most probably explains the red-roe deer mixed sequences seen in some of these samples, also after repetition of the whole sequencing process (data not shown). Red-roe deer hybrids seem biologically improbable, as they belong to two different subfamilies. Moreover, the rutting season of the two species does not match [43] and their biological parameters (i.e. height, weight) are too different, to assume the existence of red-roe deer hybrids. However, the *PRNP*-sequence is too similar among deer to be of use as a reliable tool for species identification, although some specific variations are indicative for each species. These results clearly show that anamnestic data based on phenotypical species determination must be further examined in case of doubt.

As already described in studies from Italy, Norway, Sweden, Spain and Great Britain *PRNP* in roe deer is a very conserved gene [25, 26, 47–49]. All tested animals from Germany were homozygous for wildtype (wt, T_98_P_168_Q_226_I_247_), but a small subset with a SM at codon 42. This genetic homogeneity could be explained by the long-term consequences of historic events European roe deer populations went through with an ancient population that was much smaller than it is today. For example, findings from the Mesolithic period and documentations of royal hunting events in the nineteenth century, point out that red deer and wild boar were major game species, while roe deer were hunted only occasionally and in low numbers. Furthermore, numerous wars and revolutions had a huge effect on roe deer populations, almost resulting in extinction, creating a genetic bottleneck [50]. However, beside the two Swedish animals showing a SM at codon 24 [25], we identified five German roe deer with a SM at codon 42, indicating that further *PRNP*-variation might be seen at low frequencies. With the given sample size, it can be seen, that the probability to oversee other genotypes than wt/wt is very low. Nevertheless, rare genotypes (< 1.2%) will not be found using this sample size. Yet, intensive testing would be required to prove their occurrence.

Neither the tested fallow deer nor the single WTD, reindeer, Bactrian deer and Père David’s deer lead to the identification of yet unknown polymorphisms. All eight fallow deer were of identical genotype N_138_E_226_/N_138_E_226_, which is considered wildtype for this species, just as previously described for free ranging fallow deer from Great Britain, Spain and Sweden [14]. However, due to the low number of samples, the confidence interval for the genotype N_138_E_226_/N_138_E_226_ is broad (100%). Because of the limited sample size, the results for fallow deer should be interpreted with caution. At the current confidence level of 95%, novel genotypes with an occurrence of less than 31.2% might have been overseen. Considering the historical background of Père David’s deer, its *PRNP* sequence is interesting as this species was almost extinct and therefore went through a severe genetic bottleneck that resulted in the current population being based on only 11 ancestors [35, 51, 52]. Similar observations were made for the small population of Chinese water deer (*Hydropotes inermis*) in Woburn Abbey, UK [26]. However, in contrast to roe deer it still shows *PRNP* variation, even though in low frequencies, therefore other factors must have an impact on *PRNP* conservation and diversity.

In red deer, we found three non-synonymous polymorphisms at codons 98 (T_98_/A_98_), 226 (Q_226_/E_226_) and 247 (I_247_/L_247_) as well as a novel 24 bp deletion leading to six haplotypes wt (T_98_P_168_Q_226_I_247_), E_226_, A_98_, A_98_L_247_, Δ_69-77_, Δ_69-77_A_98_ and 14 genotypes respectively. Other codons with variation such as G_59_/S_59_, P_168_/S_168_ and M_208_/I_208_ which were previously described for different red deer populations [14] were not seen in German and Danish red deer. Nevertheless, our data, showing a predominance of wt and E_226_ haplotypes in red deer, are in accordance with recently published data from Norway, Great Britain, Italy, Portugal and Spain, indicating that these haplotypes are predominantly seen across Europe [26, 47–49, 53]. In contrast, a less frequent genotype is A_98_/A_98_, which is prominent in Scotland but not in England, was mostly detected in the southern half of Germany, with distinct clusters in the middle (region 5) and southern (region 1, 2) areas. Accordingly, there are no reports of this genotype in Norway and Sweden and only low frequencies in most European countries [26, 47, 53]. Generally, A_98_ is more frequently seen in Iberian red deer (*Cervus elaphus hispanicus*), which is native to Spain, as compared to the European red deer, indigenous in the Northern parts of Europe [49]. These findings suggest a regional trend of the A_98_-genotype with higher frequencies in southern Europe, that may include the southern half of Germany. However, the number of genotyped red deer from northern countries, including those here analyzed from Denmark, are too low in comparison to those of southern European countries to draw final conclusions.

To the best of our knowledge we, together with French colleagues (Moazami-Goudarzi et al., unpublished, in preparation) are the first to report a 24 bp deletion in red deer. The loss of one octapeptide seems to be common in deer, as it has been previously observed in Chinese Water deer and reindeer [26, 54]. Red deer carrying a haplotype with deletion, either Δ_69-77_ or Δ_69-77_A_98_, almost exclusively occurred in western Germany in geographically close proximity to France. As in French red deer the same polymorphisms were detectable (Moazami-Goudarzi et al., unpublished, in preparation), the assumption of the existence of different red deer “*PRNP*-linages” seems reasonable. This theory is supported by the results observed at the German-Czech border. There, the I_247_/L_247_ heterozygous red deer were predominantly seen, matching the single homozygous animal for L_247_, which has only been described in the Czech Republic so far [26]. Further European wide investigations including a large number of individuals will be necessary to determine the existence of such clusters or regional “linages”.

The synonymous polymorphism at codon 185 (nucleotide position 555) seen in a single German red deer, is similar to one described in a study of WTD from Illinois and Wisconsin [34], but has not previously been reported in red deer. However, while WTD encoded either 555c/t, the red deer showed a 555c/a exchange. Additional synonymous polymorphisms at codons 21, 78 and 136 were found not only in our study but also throughout Europe [26, 47, 49, 53]. Although synonymous mutations do not result in amino acid substitutions, our observations and similar studies suggest a linkage of SM to non-synonymous polymorphisms [26, 47, 49, 53]. It therefore cannot be excluded that they may link to further polymorphisms e.g. in the non-coding regions of the gene, thereby possibly influencing expression levels. Therefore, silent mutations should not be neglected in studies on genotypic influence on CWD-susceptibility. Furthermore, as discussed before with *PRNP* lineages, there seem to be regional differences of SMs. For example, at codon 21 (nucleotide position 63) in Italian, German and Danish deer a 63c/t heterozygosity was seen, while Scottish deer encoded either 63c/c, 63c/g or 63 g/g [47]. Additionally, the SM at codon 78 has only been seen in our study and Italian red deer [47]. In contrast, S_168_ was seen exclusively in Scottish red deer in linkage with A_98_ (haplotype A_98_S_168_), but neither in this study nor in similar studies on *PRNP*-diversity outside UK. Hence, it is tempting to speculate of an island phenomenon for deer living in UK. In any case, the results support the hypothesis of “*PRNP*-linages” indicating a closer relationship between Italian, German, and Danish red deer.

The investigation of sika deer revealed only slight evidence of *PRNP* variation. However, due to the small number of samples, the results must be interpreted with caution, because genotypes with an occurrence of less than 7.4% might have been overseen (95% confidence level). All three sika deer showing wt/E_226_ heterozygosity derive from the same deer farm in Lower Bavaria (region 2), but two different herds. Variation in European pure sika deer have not yet been described in contrast to Korean sika deer, where the E_226_ polymorphism is common to this species [55]. However, the animals have been identified by phenotype only. Therefore, besides a novel polymorphism in this species, the existence of sika-red hybrids has to be taken into account as previously described for British sika deer [26]. Additionally, the silent mutation at codon 133 was solely seen in Bavarian samples of wild sika deer. This might not only indicate differences between populations held in captivity and free ranging deer, but also geographically clustered “*PRNP* lineages” as discussed for red deer. Natural sika deer habitats are very limited and spatially separated in Germany making the migration of individuals between populations unlikely [43]. Nevertheless, due to the low number of sika deer samples, the interpretation of these results remains difficult. Additional samples need to be analyzed.

PrP^C^ is composed of two major domains, linked by a hydrophobic region. While the N-terminal region (codons 23–123), containing the octapeptide repeats, seems intrinsically disordered, the C-terminal domain (codons 124–230) is globular structured, consisting of three α-helices and two β-strands interacting with each other. During maturation, PrP^C^ loses its first and last about 20 amino acids, which are replaced by a GPI-anchor at the C-terminal end [56]. It is assumed that amino acid substitutions may change the secondary and tertiary structure of PrP^C^, which in turn might influence its likeliness to misfold to PrP^Sc^ [57–60]. In accordance with this, analyses of genotype frequencies among North American CWD-cases revealed polymorphisms with lower CWD-prevalence than others [20, 21, 34, 61]. Additionally, transmission studies in transgenic mice or deer expressing different genotypes lead to the finding of alleles prolonging incubation periods and altering attack rates [19, 62, 63]. Based on this data first estimations on the potential influence of the PM found in our study on CWD-susceptibility will be discussed here. However, it should be borne in mind that further in vivo and in vitro investigations are necessary to evaluate their effect.

The Q_226_/E_226_ allele, which shows high variability in our red deer samples, seems promising to alter CWD-susceptibility, as it is positioned in the third α-helix of PrP^C^, a part of the protein which is thought to interact with other proteins during PrP^Sc^-formation [64]. Codon 226 is also associated with regulation of CWD-strain selection [65]. Additionally, nearby PM such as the S_225_/F_225_ in mule deer as well as the Q_222_/K_222_ in goats provide resistance to prion diseases [62, 66]. Moreover, studies in transgenic and gene targeted mice, inoculated with Finnish and North American moose CWD isolates, suggest that E_226_ restricts PrP^Sc^ propagation of Finnish moose prions [23]. However, the first CWD-case in a Norwegian red deer was homozygous for E_226_ [67], indicating that the protection, if any, is not complete. An introduction of CWD to Germany and Denmark therefore would most probably pose a threat to large proportions of indigenous deer populations, as both wt and E_226_ abundantly occur in roe, sika and red deer.

Asparagine at codon 138 (N_138_), an allele naturally occurring in all fallow deer examined to date, including the animals of our study, is thought to provide high CWD-resistance [68]. Interestingly, a study in gene-targeted mice either homozygous for N_138_ or heterozygous for S_138_/N_138_ revealed only subclinical CWD in this mouse model and low transmission efficiency in following in vitro tests [69]. However, intensive genotyping in Icelandic sheep, due to ongoing classical Scrapie outbreaks, revealed the occurrence of sheep with N_138_ heterozygosity and that they are represented less frequently among classical scrapie cases. However, in vitro results indicate, that this allele only provide insufficient protection against scrapie, as conversion in Protein Misfolding Cyclic Amplification (PMCA) was not completely inhibited [70]. Further studies in *Drosophila* transgenic for N_138_ or S_138_, revealed, that even though N_138_ prolongs incubation period of infected flies, this effect is strain dependent [71]. Therefore, if N_138_ has a notable impact on CWD susceptibility, solely or in combination with another PM (i.e. E_226_ in fallow deer) or if susceptibility is rather strain depended remains to be tested.

T_98_/A_98_ reported in this studies red deer, has also been described for South Korean sika deer and elk [55, 72]. At the same position a threonine to serine substitution (T_98_/S_98_) occurs at the same position in muntjac deer, South Korean elk and sika deer [26, 55, 72]. There are no available data on the impact of PMs at codon 98 on CWD susceptibility, but studies on nearby codons report that WTD with H_95_/Q_95_ or S_96_/S_96_ are underrepresented among CWD cases [20] and no CWD positive WTD with R_96_/R_96_ have been found so far. However, the overall numbers of the latter PM are rare, prohibiting final conclusions [34]. Further PMs nearby are found in WTD and Chinese water deer which both encode S_100_/N_100_ [73], whereas South Korean elk and sika deer show a S_100_/G_100_ variation [55, 72]. Interestingly, structural studies addressing the electrostatic potential of amino acids indicate a higher stability for the G_100_-conformer [72].

The isoleucine to leucine substitution (L_247_) is unlikely to influence CWD-susceptibility as it is within the C-terminal end of the protein, which is cleaved after prion protein maturation [74]. Additionally, the substitution of constitutional isomers, which share similar biochemical properties should neither influence the stability nor the function of the protein. Yet, unknown effects on pathogenesis and CWD-susceptibility cannot be excluded without further investigation.

To the best of our knowledge, there are no studies on the effect of deletions within the *PRNP* sequence on CWD-susceptibility, a characteristic which has not only been found in German and French red deer reported here but also in Chinese Water deer and reindeer [26, 54]. Cattle with an additional octapeptide repeat showed no alteration of susceptibility to BSE [75], but it has to be bear in mind that *PRNP*-variation do not have an effect on BSE susceptibility. Further in vivo and in vitro studies, may clarify if deletions have an impact on CWD-susceptibility.

Taken together, this study contributes to the knowledge of *PRNP* diversity in European cervid populations, including the most common deer species, native to Germany as well as several zoo animals and a small Danish red deer population. While roe and fallow deer have a highly conserved *PRNP* gene and sika deer only shows a single PM in farmed animals, red deer *PRNP* is highly variable. In total, seven haplotypes, of which six were seen in red deer, lead to 14 genotypes respectively. Based on data from North American and Northern European CWD cases, it seems most likely that the further spread of CWD would put large proportions of the German and Danish deer populations at risk. It is still to be determined by further in vitro and in vivo studies, if specific PMs, such as the T_98_/A_98_, I_247_/L_247_ and the 24 bp deletion only seen in European deer so far, influence PrP^C^ stability, hence CWD-susceptibility.

### Supplementary Information

**Additional file 1. Definition of the 12 German regions.** This map of Germany displays the 12 regions we defined along the borders of the Federal States: Germany 01 = Baden-Wuerttemberg; Germany 02 = Bavaria; Germany 03 = Rhineland-Palatinate and Saarland; Germany 04 = Hesse; Germany 05 = Thuringia; Germany 06 = Saxony; Germany 07 = North Rhine-Westphalia; Germany 08 = Bremen and Lower Saxony; Germany 09 = Saxony-Anhalt; Germany 10 = Brandenburg and Berlin; Germany 11 = Hamburg and Schleswig–Holstein; Germany 12 = Mecklenburg Western Pomerania.

**Additional file 2. Flow diagram on sample collection for genotyping German and Danish cervids.** Of the initial 3161 German and 31 Danish samples those with a grey back ground had to be excluded from statistical analyis. The blue dotted lines indicate the two steps of the workflow where samples were randomly picked for genotyping. The numbers in the green boxes show the final number of genotyped samples per species. The German map at the top left corner display the 12 regions we defined along the borders of the Federal States: Germany 01 = Baden-Wuerttemberg; Germany 02 = Bavaria; Germany 03 = Rhineland-Palatinate and Saarland; Germany 04 = Hesse; Germany 05 = Thuringia; Germany 06 = Saxony; Germany 07 = North Rhine-Westphalia; Germany 08 = Bremen and Lower Saxony; Germany 09 = Saxony-Anhalt; Germany 10 = Brandenburg and Berlin; Germany 11 = Hamburg and Schleswig–Holstein; Germany 12 = Mecklenburg Western Pomerania.

**Additional file 3. Accession numbers of sequences used as reference for species determination.**

**Additional file 4. Accession numbers of sequences used as reference for this study.**

**Additional file 5. List of R packages used to create the figures and tables.**

**Additional file 6.Hunting bag on county level for the hunting season 2021/22.** Number of the hunted animals is indicated with the blue colour scheme. The darker the colour, the more animals were hunted. Counties coloured in white-blue are not inhabited by the corresponding species. For red deer, most of those counties are within the red-deer-free districts, where red deer are not allowed to live and thereby were shot at sight. This figure was used to compare the location of the samples collected for the study to the annually hunting bag to identify overrepresented areas and counties where no sampling was possible.

**Additional file 7. Prevalence and confidence intervals of genotypes in Germany and Denmark.**

## Data Availability

The datasets used and/ or analyzed during the current study are available from the corresponding author on reasonable request.
