# Supplementary figures and images for: Prion protein gene (PRNP) variation in German and Danish cervids
Source: Vet Res. 2024 Aug 2;55:98. doi: 10.1186/s13567-024-01340-8 (PMC11297704; doi:10.1186/s13567-024-01340-8)

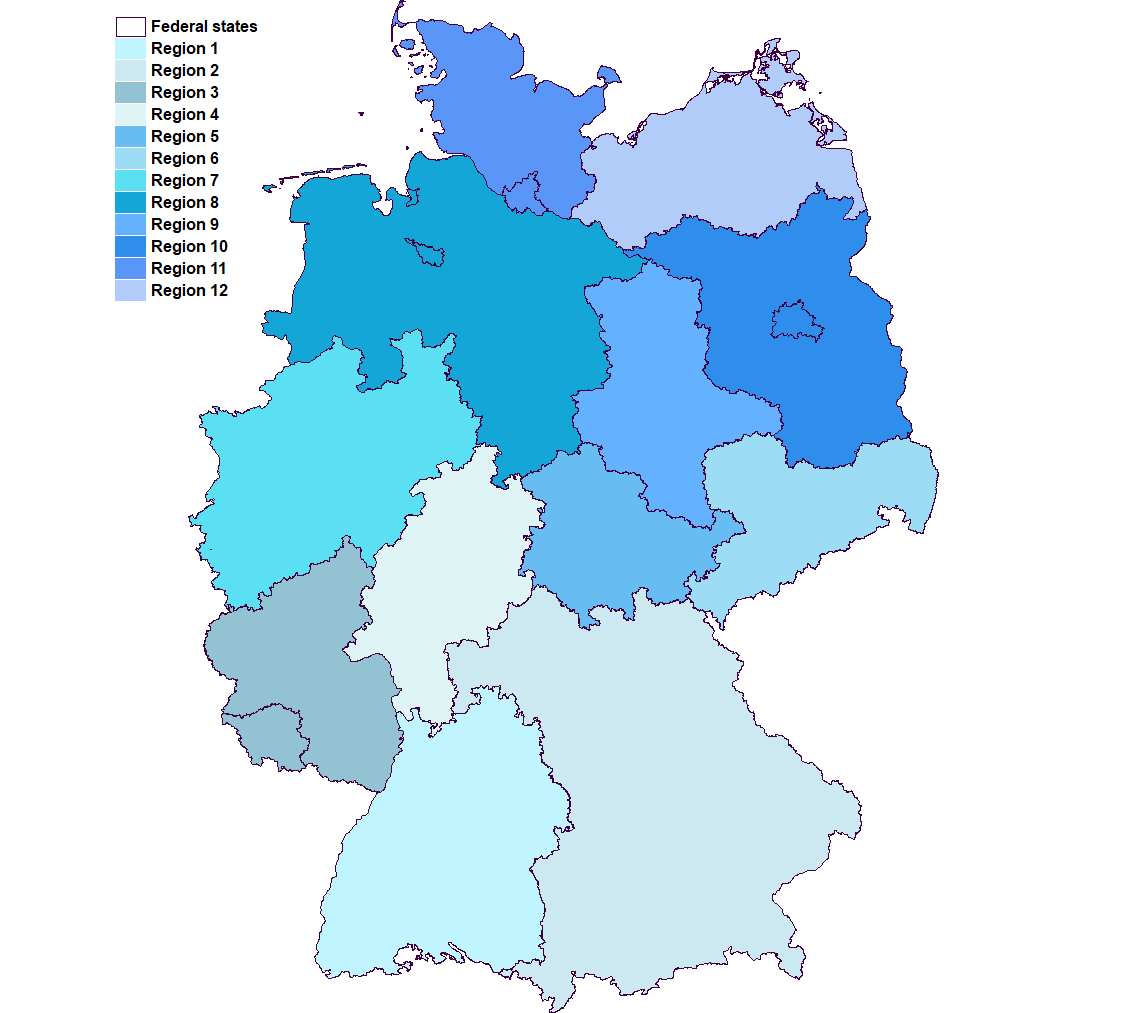


2

1

3

4

5

6

8

7

10

12

11

9

Supplement: Supplementary file 1 — Additional file 1. Definition of the 12 German regions. This map of Germany displays the 12 regions we defined along the borders of the Federal States: Germany 01 = Baden-Wuerttemberg; Germany 02 = Bavaria; Germany 03 = Rhineland-Palatinate and Saarland; Germany 04 = Hesse; Germany 05 = Thuringia; Germany 06 = Saxony; Germany 07 = North Rhine-Westphalia; Germany 08 = Bremen and Lower Saxony; Germany 09 = Saxony-Anhalt; Germany 10 = Brandenburg and Berlin; Germany 11 = Hamburg and Schleswig–Holstein; Germany 12 = Mecklenburg Western Pomerania. [file 13567_2024_1340_MOESM1_ESM.docx]

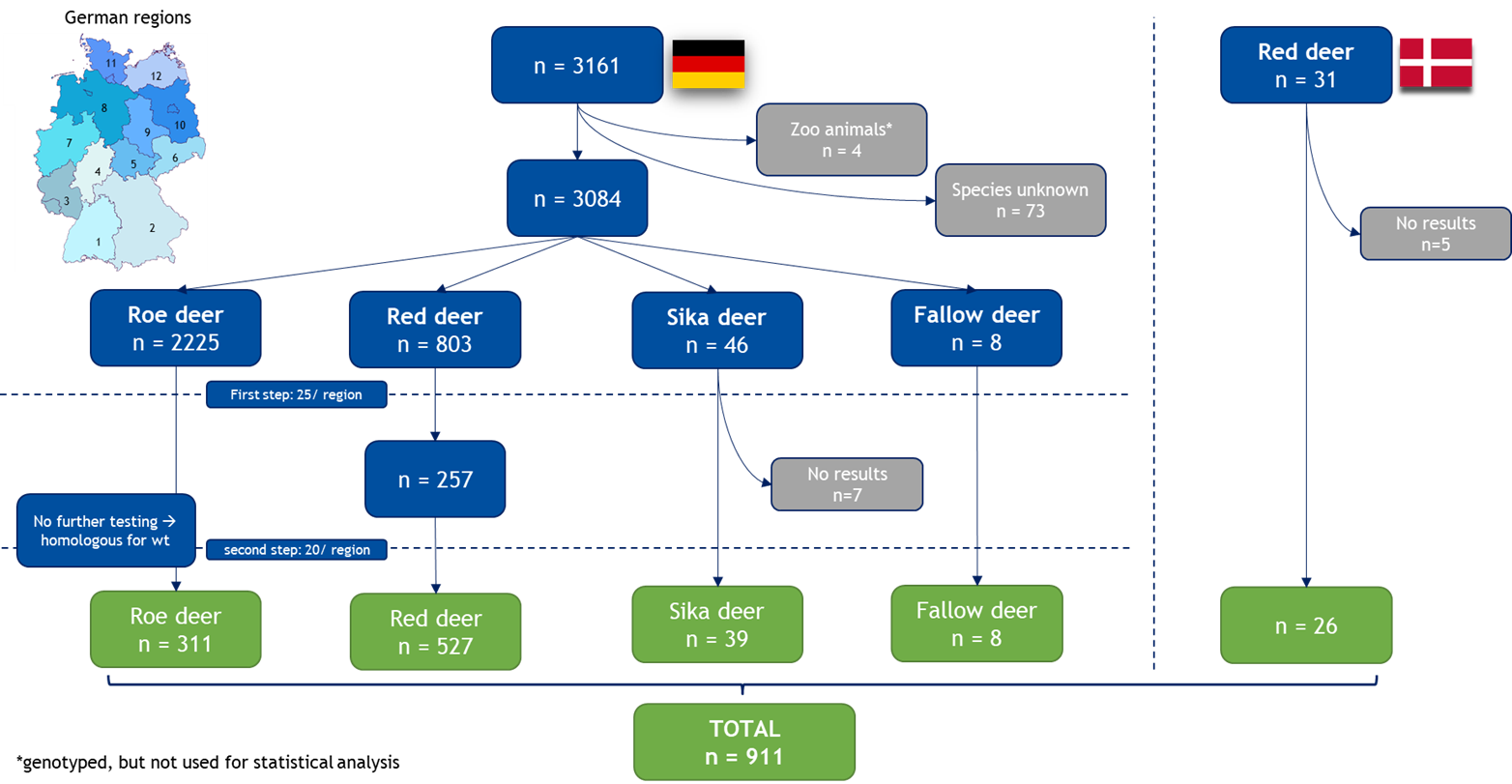

Supplement: Supplementary file 2 — Additional file 2. Flow diagram on sample collection for genotyping German and Danish cervids. Of the initial 3161 German and 31 Danish samples those with a grey back ground had to be excluded from statistical analyis. The blue dotted lines indicate the two steps of the workflow where samples were randomly picked for genotyping. The numbers in the green boxes show the final number of genotyped samples per species. The German map at the top left corner display the 12 regions we defined along the borders of the Federal States: Germany 01 = Baden-Wuerttemberg; Germany 02 = Bavaria; Germany 03 = Rhineland-Palatinate and Saarland; Germany 04 = Hesse; Germany 05 = Thuringia; Germany 06 = Saxony; Germany 07 = North Rhine-Westphalia; Germany 08 = Bremen and Lower Saxony; Germany 09 = Saxony-Anhalt; Germany 10 = Brandenburg and Berlin; Germany 11 = Hamburg and Schleswig–Holstein; Germany 12 = Mecklenburg Western Pomerania. [file 13567_2024_1340_MOESM2_ESM.docx]

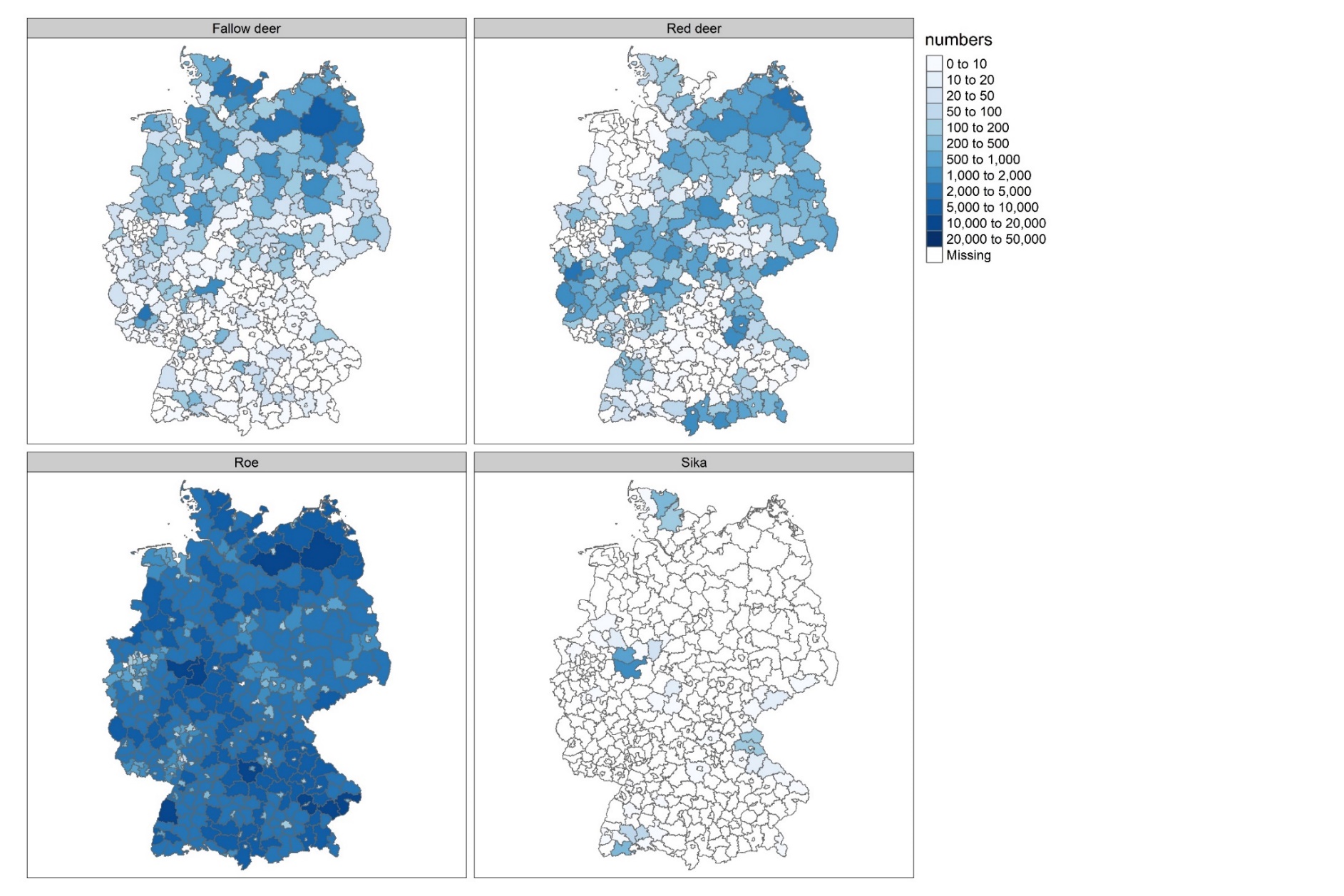

Supplement: Supplementary file 6 — Additional file 6.Hunting bag on county level for the hunting season 2021/22. Number of the hunted animals is indicated with the blue colour scheme. The darker the colour, the more animals were hunted. Counties coloured in white-blue are not inhabited by the corresponding species. For red deer, most of those counties are within the red-deer-free districts, where red deer are not allowed to live and thereby were shot at sight. This figure was used to compare the location of the samples collected for the study to the annually hunting bag to identify overrepresented areas and counties where no sampling was possible. [file 13567_2024_1340_MOESM6_ESM.docx]
