# Supplementary material for: Prion protein gene (PRNP) variation in German and Danish cervids
Source: Vet Res. 2024 Aug 2;55:98. doi: 10.1186/s13567-024-01340-8 (PMC11297704; doi:10.1186/s13567-024-01340-8)
Supplement: Supplementary file 3 — Additional file 3. Accession numbers of sequences used as reference for species determination. [file 13567_2024_1340_MOESM3_ESM.docx]

**Additional file 3 - Accession numbers of sequences used as reference for species determination**

| **Species** | **Accession number** |
| --- | --- |
| Cattle (Bos taurus) | NC_006853.1 |
| Sheep (Ovis aries) | NC_001941.1, DQ903224.1, OM869878.1 |
| Goat (Capra hircus) | NC_005044.2 |
| Western roe deer (Capreolus capreolus) | NC_020684.1, KJ558314.1, KJ681482.1, KX550268.1 |
| Eastern roe deer (Capreolus pygargus) | NC_025271.1 |
| Red deer (Cervus elaphus) | NC_007704.2, MN746793.1, MT266663.1, KC562172.1, KY313815.1, MF872249.1, MF872248.1 |
| Iberian red deer (Cervus e. hispanicus) | AF489281.1 |
| Sika deer (Cervus nippon) | NC_006993.1 |
| Moose (Alces alces) | NC_020677.1 |
| Fallow deer (Dama dama) | NC_020700.1 |
| White-tailed deer (Odocoileus virginianus) | NC_015247.1 |
| Barasingha (Rucervus duvaucelii) | NC_020743.1 |
| Reindeer (Rangifer tarandus) | NC_007703.1 |
| Wapiti (Cervus canadensis) | NC_050863.1 |
| Altai wapiti (Cervus c. sibiricus) | ON677348.1 |
| Père David’s deer (Elaphurus davidianus) | NC_018358.1 |
| Sambar (Rusa unicolor) | NC_031835.1 |
