# Supplementary material for: Prion protein gene (PRNP) variation in German and Danish cervids
Source: Vet Res. 2024 Aug 2;55:98. doi: 10.1186/s13567-024-01340-8 (PMC11297704; doi:10.1186/s13567-024-01340-8)
Supplement: Supplementary file 4 — Additional file 4. Accession numbers of sequences used as reference for this study. [file 13567_2024_1340_MOESM4_ESM.docx]

**Additional file 4 Accession numbers of sequences used as reference for this study**

| **Accession number** | **Species** | **Reference** |
| --- | --- | --- |
| DQ154293 | Reindeer (*R. tarandus*) | [33] |
| MG856905 | White-tailed deer *(O. virginianus)* | [34] |
| MK103016 | Roe deer (*C. capreolus*) | [26] |
| MK103017 | Fallow deer (*Dama dama*) | [26] |
| MK103018 | Sika deer (*C. nippon*) | [26] |
| MK103027 | Red deer (*C. elaphus*) | [26] |
| MW804583 | Père David’s deer *(E. davidianus)* | [35] |
