# Supplementary material for: Prion protein gene (PRNP) variation in German and Danish cervids
Source: Vet Res. 2024 Aug 2;55:98. doi: 10.1186/s13567-024-01340-8 (PMC11297704; doi:10.1186/s13567-024-01340-8)
Supplement: Supplementary file 5 — Additional file 5. List of R packages used to create the figures and tables. [file 13567_2024_1340_MOESM5_ESM.docx]

**Additional file 5 List of R packages used to create the figures and tables**

| **Package** | **Category** | **Authors** | **Title** | **Year** | **Journal/ Publisher** | **Volume** | **Number** | **Pages** | **DOI/ISBN** | **URL** |
| --- | --- | --- | --- | --- | --- | --- | --- | --- | --- | --- |
| **sf2** | Article | Edzer Pebesma | Simple Features for R: Standardized Support for Spatial Vector Data | 2018 | The R Journal | 10 | 1 | 439-446 | 10.32614/RJ-2018-009 | https://doi.org/10.32614/RJ-2018-009 |
| **tmap** | article | Martijn Tennekes | tmap: Thematic Maps in R | 2018 | Journal of Statistical Software | 84 | 6 | 1--39 | 10.18637/jss.v084.i06 | (Leer) |
| **sf** | book | Edzer Pebesma, Roger Bivand | Spatial Data Science: With applications in R | 2023 | Chapman and Hall/CRC |  |  |  | 10.1201/9780429459016 | https://r-spatial.org/book/ |
| **ggplot2** | book | Hadley Wickham | ggplot2: Elegant Graphics for Data Analysis | 2016 | Springer-Verlag New York |  |  |  | 978-3-319-24277-4 | https://ggplot2.tidyverse.org |
| **survival2** | book | Terry M. Therneau, Patricia M. Grambsch | Modeling Survival Data: Extending the {C}ox Model | 2000 | Springer |  |  |  | 0-387-98784-3 | (Leer) |
| **tidyr** | manual | Hadley Wickham, Davis Vaughan, Maximilian Girlich | tidyr: Tidy Messy Data | 2023 |  |  |  |  |  | https://CRAN.R-project.org/package=tidyr |
| **readxl** | manual | Hadley Wickham, Jennifer Bryan | readxl: Read Excel Files | 2023 |  |  |  |  |  | https://CRAN.R-project.org/package=readxl |
| **purrr** | manual | Hadley Wickham, Lionel Henry | purrr: Functional Programming Tools | 2023 |  |  |  |  |  | https://CRAN.R-project.org/package=purrr |
| **dplyr** | manual | Hadley Wickham, Romain François, Lionel Henry, Kirill Müller, Davis Vaughan | dplyr: A Grammar of Data Manipulation | 2023 |  |  |  |  |  | https://CRAN.R-project.org/package=dplyr |
| **epiR** | manual | Mark Stevenson, Evan Sergeant | epiR: Tools for the Analysis of Epidemiological Data | 2023 |  |  |  |  |  | https://CRAN.R-project.org/package=epiR |
| **base** | manual | R Core Team | R: A Language and Environment for Statistical Computing | 2023 |  |  |  |  |  | https://www.R-project.org/ |
| **datasets** | manual | R Core Team | R: A Language and Environment for Statistical Computing | 2023 |  |  |  |  |  | https://www.R-project.org/ |
| **graphics** | manual | R Core Team | R: A Language and Environment for Statistical Computing | 2023 |  |  |  |  |  | https://www.R-project.org/ |
| **grDevices** | manual | R Core Team | R: A Language and Environment for Statistical Computing | 2023 |  |  |  |  |  | https://www.R-project.org/ |
| **methods** | manual | R Core Team | R: A Language and Environment for Statistical Computing | 2023 |  |  |  |  |  | https://www.R-project.org/ |
| **stats** | manual | R Core Team | R: A Language and Environment for Statistical Computing | 2023 |  |  |  |  |  | https://www.R-project.org/ |
| **utils** | manual | R Core Team | R: A Language and Environment for Statistical Computing | 2023 |  |  |  |  |  | https://www.R-project.org/ |
| **survival** | manual | Terry M Therneau | A Package for Survival Analysis in R | 2023 |  |  |  |  |  | https://CRAN.R-project.org/package=survival |
