# Supplementary material for: Prion protein gene (PRNP) variation in German and Danish cervids
Source: Vet Res. 2024 Aug 2;55:98. doi: 10.1186/s13567-024-01340-8 (PMC11297704; doi:10.1186/s13567-024-01340-8)
Supplement: Supplementary file 7 — Additional file 7. Prevalence and confidence intervals of genotypes in Germany and Denmark. [file 13567_2024_1340_MOESM7_ESM.docx]

**Additional file 7 Prevalence and confidence intervals of genotypes in Germany and Denmark**

| **Species** | **Country** | **GT** | **Freq (%)** | **samples** | **Proportion** | **Confidence interval** | |
| --- | --- | --- | --- | --- | --- | --- | --- |
|  |  |  |  |  |  | **lower** | **upper** |
| Fallow deer | Germany | N_138_E_226_/N_138_E_226_ | 8 | 8 | 100,0 | 63.1 | 100.0 |
| **Fallow deer** | **Germany** | **all** |  | **8** | **100.0** |  |  |
| Red deer | Denmark | A_98_/E_226_ | 6 | 26 | 23.1 | 9.0 | 43.6 |
| Red deer | Denmark | E_226_/E_226_ | 17 | 26 | 65.4 | 44.3 | 82.8 |
| Red deer | Denmark | wt/E_226_ | 2 | 26 | 7.7 | 0.9 | 25.1 |
| Red deer | Denmark | wt/wt | 1 | 26 | 3.8 | 0.1 | 19.6 |
| **Red deer** | **Denmark** | **all** |  | **26** | **100.0** |  |  |
| Red deer | Germany | A_98_/A_98_ | 16 | 527 | 3.0 | 1.7 | 4.9 |
| Red deer | Germany | A_98_/A_98_L_247_ | 2 | 527 | 0.4 | 0.0 | 1.4 |
| Red deer | Germany | A_98_/wt | 40 | 527 | 7.6 | 5.5 | 10.2 |
| Red deer | Germany | E_226_/A_98_ | 91 | 527 | 17.3 | 14.1 | 20.8 |
| Red deer | Germany | E_226_/A_98_L_247_ | 4 | 527 | 0.8 | 0.2 | 1.9 |
| Red deer | Germany | E_226_/E_226_ | 210 | 527 | 39.8 | 35.6 | 44.2 |
| Red deer | Germany | wt/A_98_L_247_ | 2 | 527 | 0.4 | 0.0 | 1.4 |
| Red deer | Germany | wt/E_226_ | 99 | 527 | 18.8 | 15.5 | 22.4 |
| Red deer | Germany | wt/wt | 51 | 527 | 9.7 | 7.3 | 12.5 |
| Red deer | Germany | Δ_69-77_/E_226_ | 3 | 527 | 0.6 | 0.1 | 1.7 |
| Red deer | Germany | Δ_69-77_/wt | 1 | 527 | 0.2 | 0.0 | 1.1 |
| Red deer | Germany | Δ_69-77_A_98_/A_98_ | 2 | 527 | 0.4 | 0.0 | 1.4 |
| Red deer | Germany | Δ_69-77_A_98_/E_226_ | 4 | 527 | 0.8 | 0.2 | 1.9 |
| Red deer | Germany | Δ_69-77_A_98_/wt | 2 | 527 | 0.4 | 0.0 | 1.4 |
| **Red deer** | **Germany** | **all** | **527** | **527** | **100.0** |  |  |
| Roe deer | Germany | wt/wt | 311 | 311 | 100.0 | 98.8 | 100.0 |
| **Roe deer** | **Germany** | **all** | **311** | **311** | **100.0** |  |  |
| Sika deer | Germany | wt/E_226_ | 3 | 39 | 7.7 | 1.6 | 20.9 |
| Sika deer | Germany | wt/wt | 36 | 39 | 92.3 | 79.1 | 98.4 |
| **Sika deer** | **Germany** | **all** | **39** | **39** | **100.0** |  |  |
